# Determinants of viral load non-suppression among adolescents in Mbale District, Eastern Rural Uganda

**DOI:** 10.1186/s12981-021-00408-1

**Published:** 2021-12-04

**Authors:** Joel Maena, Aduragbemi Banke-Thomas, Nelson Mukiza, Cynthia Ndikuno Kuteesa, Ronald Makanga Kakumba, Hajira Kataike, Samuel Kizito, Juliet Allen Babirye, Rita Nakalega

**Affiliations:** 1 Makerere University-Johns Hopkins University (MU-JHU) Research Collaboration Kampala, Kampala, Uganda; 2 Department of Public Health and Preventive Medicine, School of Medicine, University of Liverpool, Liverpool, UK; 3 RineCynth Advisory Limited, Kampala, Uganda; 4 MRC/UVRI & LSHTM Uganda Research Unit, Entebbe, Uganda; 5 Department of Global Health, School of Public Health, Boston University, Boston, USA

**Keywords:** HIV, ART, Viral Load, Adolescents, ART, Non-suppression, Uganda

## Abstract

**Background:**

Adolescents are lagging behind in the “third 95” objective of the Joint United Nations Program on HIV/AIDS requiring 95% of individuals on antiretroviral therapy (ART) to have viral load (VL) suppression. This study aimed to describe factors associated with viral non-suppression among adolescents in Mbale district, Uganda.

**Methods:**

We conducted a retrospective review of routinely collected HIV programme records. Data such as age, education, ART Regimen, ART duration, WHO Clinical stage, comorbidities, etc., were extracted from medical records for the period January 2018 to December 2018. Descriptive analysis was done for continuous variables using means and frequencies to describe study sample characteristics, and to determine the prevalence of outcome variables. We used logistic regression to assess factors associated with VL non-suppression among adolescents.

**Results:**

The analysis included 567 HIV-infected adolescents, with 300 (52.9%) aged between 13 to 15 years, 335 (59.1%) female, and mean age of 15.6 years (interquartile range [IQR] 13.5–17.8. VL non-suppression was 31.4% (178/567). Male sex (AOR = 1.78, 95% CI 1.06, 2.99; *p* < 0.01), age 16–19 years (AOR = 1.78, 95% CI 1.06, 2.99; *p* < 0.05), No formal education (AOR = 3.67, 95% CI 1.48–9.09; *p* < 0.01), primary education (AOR = 2.23, 95% CI 1.05–2.32; *p* < 0.01), ART duration of > 12 months to 5 years (AOR = 3.20, 95% CI 1.31–7.82; *p* < 0.05), ART duration > 5 years (AOR = 3.47, 95% CI 1.39– 8.66; *p* < 0.01), WHO Clinical Stage II (AOR = 0.48, 95% CI: 0.28, 0.82; *p* < 0.01), second-line ART regimen (AOR = 2.38, 95% CI 1.53–3.72; *p* < 0.001) and comorbidities (AOR = 3.28, 95% CI 1.20–9.00; *p* < 0.05) were significantly associated with viral non-suppression**.**

**Conclusions:**

VL non-suppression among adolescents was almost comparable to the national average. VL non-suppression was associated with being male, age 16–19 years, education level, duration on ART therapy, WHO Clinical Staging II, second-line ART regimen, and presence of comorbidities. Adolescent-friendly strategies to improve VL suppression e.g. peer involvement, VL focal persons to identify and actively follow-up non-suppressed adolescents, patient education on VL suppression and demand creation for ART are needed, especially for newly-initiated adolescents and adolescents on ART for protracted periods, to foster attainment of the UNAIDS 95–95–95 targets.

## Introduction

Globally, as of 2020, of all people living with HIV, 84% knew their status, 73% were accessing treatment and 66% were virally suppressed in 2020 [1]. While this shows improvement, this progress is highly unequal, because Sub-Saharan Africa is lagging far behind with adolescents carrying the burden of viral-load non-suppression [1]. Adolescents, especially young women and girls in sub-Saharan Africa, continue to be disproportionately affected, contributing the largest part of the overall HIV non-suppression prevalence of 47% [2]. In Uganda, six years since the operationalization of viral load monitoring, viral suppression among adolescents is still sub-optimal, at 73.7%. However, information on viral load suppression among adolescents remains scarce in Sub-Saharan Africa [3].

Viral suppression is critical in HIV treatment success, ensuring a decrease AIDS-associated morbidity and mortality, and reduction in risk of viral transmission. Low- and middle-income countries, define viral suppression as a viral load (VL) < 1000 copies/ml. It is an indicator of ART effectiveness and treatment adherence. WHO defines 'Adolescents' as individuals in the 10–19 years age group [4]. Follow-up of virological suppression status among adolescents in crucial for quick identification of treatment failure, of patients in need of intensive adherence counseling, and prevents occurrence of drug resistance, leading to unwanted switch to costly and limited ART regimens [3].

The “third 95” of the UNAIDS 95/95/95 strategy to alleviate the epidemic seeks to have 95% of the HIV-infected individuals on ART virally suppressed by 2025 [2]. The “third 95” is critical in the battle to defeat HIV/AIDS because virally suppressed individuals can barely transmit the virus [5]. Unfortunately, as of 2020, merely 66% of PLHIV on ART were virally suppressed worldwide, with adolescents contributing a bulk of the unsuppressed [6].

As of 2017, only 70% of the HIV-infected in Uganda are virally suppressed, with children, adolescents, and young adults making up about 65% of the unsuppressed group [7]. Furthermore, due to the common mode of age disaggregation employed in Uganda where adolescents between 13 and 15 are grouped with children aged 0–15 while those aged 16–19 are considered adults, specific data on adolescent VL suppression is limited [7]. Consequently, while adolescents contribute a substantial number of virally non-suppressed patients, there is limited data on the viral outcomes of this demographic [8].

Moreso, adolescents face distinctive challenges in taking ART such as normal juvenile forgetfulness and failure to take such issues seriously, disclosure concerns, and stigma, all contributing to high non-suppression rates [9]. Likewise, until recently, there has been a paucity of adolescent-friendly services and research, leading to under-diagnosis of, and disregard for, their unique challenges causing high non-suppression rates [10]. The lack of adolescent-centric HIV research has contributed to a deficiency of data on the determinants of VL non-suppression among adolescents. The very few papers available used data from the largely urbanized central region, ignoring rural societies in the East such as Mbale district [8].

Additionally, non-suppressed adolescents are more prone to HIV-related morbidity and mortality that could be circumvented through IAC and support [11]. Also, IAC mitigates subpar adherence that is common among adolescents and leads to drug-resistant HIV strains, which require expensive second-line or third-line treatments [12].

The influence of various determinants of VL non-suppression varies among populations, age groups, and settings, requiring contextual data to inform remedial measures among adolescents [13]. Therefore, understanding the distinct determinants of VL non-suppression among adolescents will give all stakeholders new insight into HIV infection among adolescents. It will further motivate adolescents to adhere to medication, thus sustaining the relevance of affordable first-line treatments by preventing drug resistance and also and help attainment of the third UNAIDS 95–95–95 target [2, 14]. This study aimed to describe factors associated with non-viral suppression among adolescents in Mbale district, Uganda.

## Methods

### Study design

We conducted a retrospective review of routinely collected data that employed methods of secondary data analysis of routinely available program data on HIV-infected adolescents receiving ART in Mbale district for the period of January and December 2018.

### Study setting

**Fig. 1:**
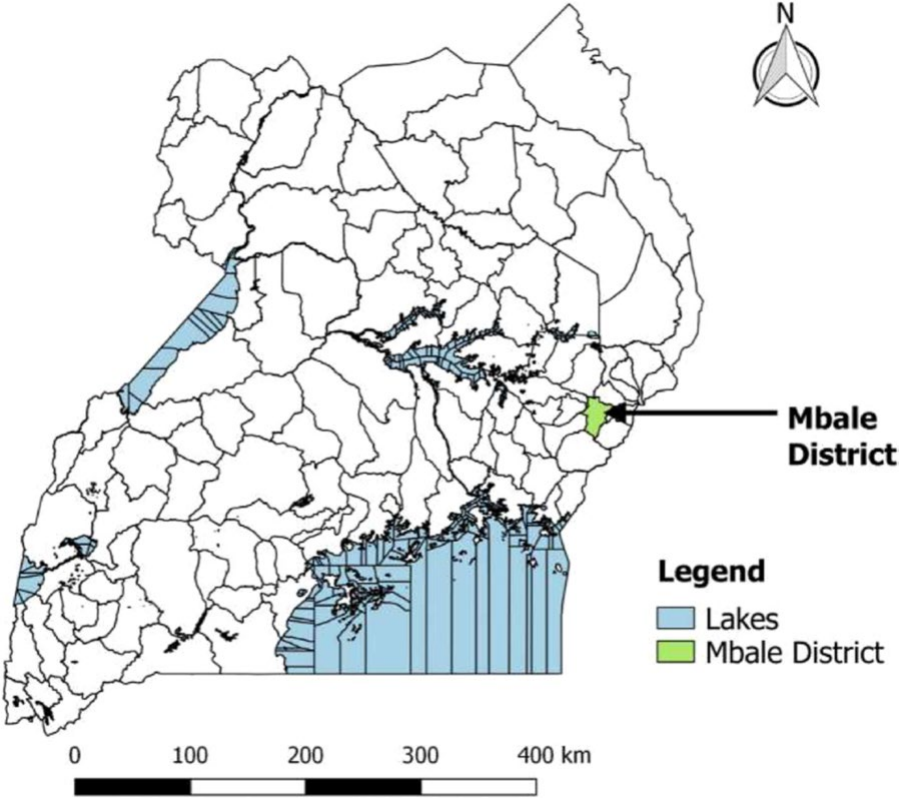
Showing Mbale District

**Fig. 2:**
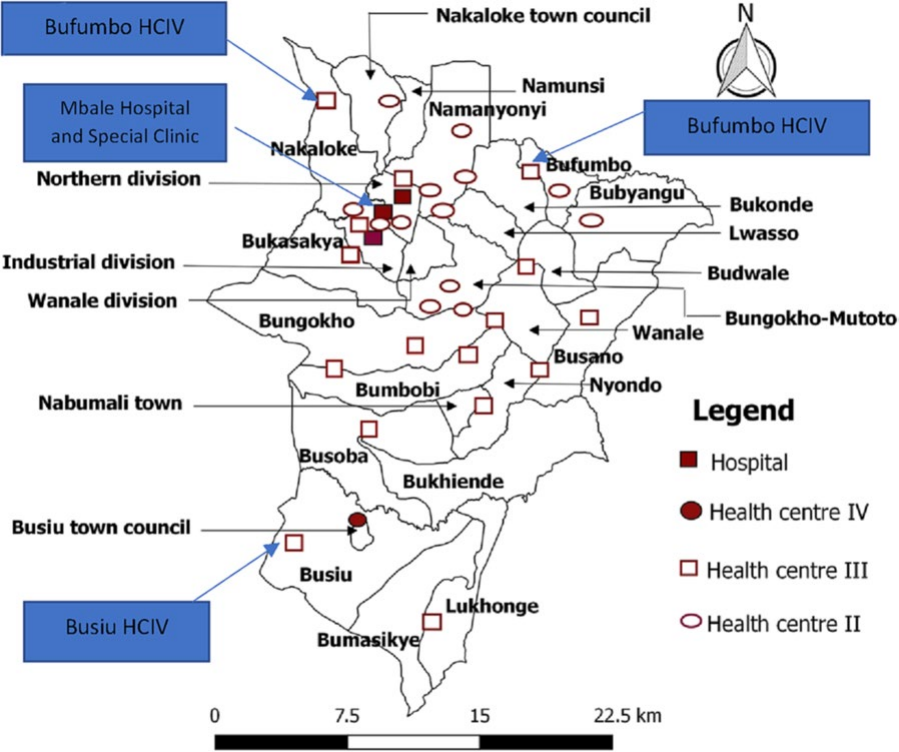
Refer to blue arrows for health centres in the study

Mbale district (Fig. 1) [15] in Eastern Uganda boasts a population of 488,960 as of 2016. 51% are female, and over 90% of the population is rural. Mbale District is a largely impoverished district with a high HIV prevalence. While the general prevalence of HIV in Mbale is 5.3%, that of adolescents is 7% [16]. Primary care facilities in Uganda are organized by administrative division and include Health Center II (parish), Health Center III (sub-county), and Health Center IV (county). Mbale has numerous health facilities, including special clinics, HCIII, HCIV, and the regional referral hospital providing HIV services, backed by the government and private funders (Fig. 2) [15, 17]. In Uganda, since 2016, the test and treat strategy is employed for all newly HIV diagnosed persons as per the WHO recommendations. VL testing is performed after 6 months following ART initiation and every 6 months for those that have HIV VL suppression. At the enrollment into the HIV care clinics, socio-demographic data on adolescents is recorded on client treatment cards (blue card), and clinical history is also documented on these cards at each visit thereafter. VL test results received from the national laboratory center for each client who had a blood sample drawn for VL testing are recorded in viral load registers and blue cards by health facility staff and provided to adolescents at subsequent clinic visits [18]. HIV-positive adolescents at five purposively selected high-volume health centers of different levels (regional referral hospital, HCIV, HCIII, special clinic), and located in different geographical regions of the district were included in the study [16]. Because of the health center selection criteria, it transpired that the higher-level centres had higher volumes and thus would provide a large number of potential study participants. Furthermore, these higher level centres serve large catchment areas, coupled with patients or adolescents from far-flung areas of the district. On that basis, we could not ignore them for smaller health centres (which also usually have challenged of doing viral load tests), thus, we ended up with 5 health centres at three health centre levels.

### Study population

We included all adolescents aged 13 to 19, on ART for at least 6 months, and had VL tests at the selected facilities. We excluded those with an unknown duration on ART, those on ART for less than six months, and those who had no VL test results. After calculation of the sample size of 383 adolescents required for the study using the Leslie Kish equation for cross-sectional studies [19] sample size equation for cross-section studies, the proportionate allocation method was used to determine the number of participants to be randomly selected from each health facility [20] Socio-demographic and clinical data including age, sex, level of education, ART Regimen, ART duration, WHO Clinical stage, comorbidities were analyzed.

### Ethical considerations

A district support letter was secured prior to attainment of local ethical approval from a local Research Ethics Committee (Mildmay Uganda REC; #REC REF 08,010-2019) and then from the University of Liverpool (Reference Number H00065262). Anonymized data was sent through a secure e-mail address. The raw data will be securely retained for five years, then discarded. The investigator was aware of the sensitive nature of the study, and that the necessary data for the study pertained to a vulnerable group. However, the anonymized data and the lack of access to participant files fully protected the group from any risk of breach of confidentiality.

### Data collection

Data collection occurred over 2 weeks in January 2020 after obtaining all approvals. Data were abstracted from the sources below;

- Paper registers: The VL registers
- Electronic ART registers where applicable
- Client treatment record (blue card) at all the health facilities.

Initially, a list of all adolescents on ART for at least 6 months at each facility was generated from the electronic database or entered into a Microsoft® Excel database 2007 version for health facilities that lacked electronic records. Each patient was given a unique study code by the facility staff, and the list of codes from each health facility was sent to the study investigator who then randomly selected the number of specified participants for each facility. The list of codes of selected participants was then posted back to health facility staff who linked the codes to participants whose records were eventually abstracted.

The health facility staff were trained on the specific indicators to be entered into the excel sheet, and the investigator visited the health facilities during the data collection process to oversee and ensure quality of the process. The study team reviewed all the records of the participants that were abstracted to ensure data quality, and corrections were performed on records with observed data errors.

Data on key demographic variables such as age and sex were abstracted from the registers and blue cards into the excel sheet. Data on the start date of ART was used to calculate the duration on ART at the time of the VL test. The date of VL testing, the date VL test results were received at the facility, and the date VL test results provided to the adolescents were all updated in a Microsoft® Excel database.

### Data analysis

Data analysis was conducted based on the primary outcome of interest in the study using the Statistical Package for the Social Sciences (SPSS) version 20.0 (IBM Corp. Armonk, NY, USA). The primary outcome “VL non-suppression” was defined as an adolescent having a VL load above the WHO-recommended detectable threshold of 1000 copies/ml after six months on ART [21]. Socio-demographic variables like age, sex, level of education, and clinical variables like ART regimen, comorbidities, or duration on ART, served as independent variables. Descriptive analysis was done for continuous variables using means depending on the normality of the distribution of the variables, and frequencies to describe the characteristics of the study sample and to determine the prevalence (proportions) of the outcome variables. We used a multivariable logistic regression model to assess factors related to VL non-suppression. Pearson’s chi-square test was used to test relationships between categorical variables. Factors with p ≤ 0.2 in bivariate analyses were included in the multivariable model. p-values of ≤ 0.05 were considered statistically significant.

## Results

We enrolled a total of 567 adolescents, more than half of the adolescents (52.9% /300) were aged between 13 and 15 years, and the mean age of the study participants was 15.6 ± 2.10. 335 (59.1%) of the participants were female. The majority of the study participants (336 or 59.3%) had attained primary education. Furthermore, 482 (85%) of the adolescents received ART services from the regional referral hospital. The mean duration on ART therapy for the study participants was 74.3 ± 41.7 months, with the largest proportion of the participants (323 or 57%) on ART therapy for over 5 years. The majority of participants (414 or 73%) were receiving 1st line of the ART regimen, and the presence of comorbidities was observed among 19 participants. About half of the adolescents (279 or 49.3%) were in WHO Clinical disease stage II (refer to Table 1).

**Table 1:** Participant characteristics

Characteristic	N (%)	Non-suppressed group N (%)
Age categories		
13–15 years	300 (52.9)	96 (32.0)
16–19 years	267 (47.1)	82 (30.7)
Sex		
Female	335 (59.1)	89 (26.6)
Male	231 (40.8)	89 (38.5)
Education level		
Secondary/Tertiary Level	200 (35.2)	49 (24.5)
Primary education level	336 (59.3)	113 (33.6)
No formal education	31 (5.5)	16 (51.6)
Health Facility Level		
Health Center III	27 (4.8)	153 (31.7)
Health Center IV	58 (10.2)	16 (27.6)
Regional Referral Hospital	482 (85)	9 (33.3)
Duration on ART		
6–12 months	59 (10.4)	9 (15.5)
> 12 months-5 years	185 (32.6)	54 (29.2)
> 5 years	323 (57)	115 (35.6)
WHO Clinical disease stage		
Stage I	132 (23.3)	42 (31.8)
Stage II	279 (49.3)	73 (26.2)
Stage III	120 (21.2)	50 (41.7)
Stage IV	35 (6.2)	13 (37.1)
ART regimen		
1st line regimen	414 (73)	107 (25.9)
2nd line regimen	150 (26.5)	68 (45.3)
3rd line regimen	3 (0.5)	
Presence of comorbidities		
Yes	19 (3.4)	167 (30.6)
No	546 (96.6)	11 (57.9)

Out of the total 567 adolecents enrolled in the study, 178 (31.4%) had VL load non-suppression. Being male (odds ratio [OR] = 1.21; 95% CI 1.12, 2.48; p ≤ 0.01), lacking formal education (OR = 3.29; 95% CI 1.51, 7.13; p ≤ 0.01), having co-morbidities (OR = 3.12; 95% CI 1.23, 7.89; p ≤ 0.05), being on second line ART regimen (OR = 2.38; 95% CI 1.61, 3.51; p ≤ 0.001) were significantly associated with VL non-suppression in univariate analysis (Table 2). Similary, adolescents on ART > 5 years and those on ART for > 12 months- < 5 years were three times (OR = 3.07; 95% CI 1.46, 6.47 p ≤ 0.05 < 0.01) and over two times (OR = 2.29; 95% CI 1.05, 4.98; p ≤ 0.01), respectively, more likely to be non-suppressed than those on ART for less than a year.

**Table 2:** Correlates of VL non-suppression

Characteristic	Bivariate analysis		Multivariate analysis	
	OR (95% CI)	p value	AOR (95% CI)	p-value
Age categories				
13–15 years	1		1	
16–19 years	0.94 (0.66,1.34)	0.741	1.78 (1.06,2.99)	< 0.05
Sex				
Female	1		1	
Male	1.21 (1.12,2.48)	< 0.01	1.86 (1.25,2.75)	< 0.01
Education level				
Secondary/Tertiary Level	1		1	
Primary education level	1.56 (1.05,2.32)	< 0.05	2.23 (1.28,3.88)	< 0.01
No formal education	3.29 (1.51,7.13)	< 0.01	3.67 (1.48,9.09)	< 0.01
Health Facility Level				
Health Center III	1			
Health Center IV	0.76 (0.28,2.04)	0.589		
Regional Referral Hospital	0.93 (0.41,2.12)	0.863		
Duration on ART				
6–12 months	1		1	
> 12 months-5 years	2.29 (1.05,4.98)	< 0.05	3.2 (1.31,7.82)	< 0.05
> 5 years	3.07 (1.46,6.47)	< 0.01	3.47 (1.39,8.66)	< 0.01
WHO Clinical disease stage				
Stage I	1		1	
Stage II	0.76 (0.48,1.19)	0.234	0.48 (0.28,0.82)	< 0.01
Stage III	1.53 (0.91,2.56)	0.106	1.03 (0.56,1.92)	0.914
Stage IV	1.27 (0.58,2.75)	0.552	0.71 (0.27,1.81)	0.475
ART regimen				
1st line regimen	1		1	
2nd line regimen	2.38 (1.61,3.51)	< 0.001	2.38 (1.53,3.72)	< 0.001
3rd line regimen				
Presence of comorbidities				
Yes	1		1	
No	3.12 (1.23,7.89)	< 0.05	3.28 (1.20,9.00)	< 0.05

In the adjusted model, being male (adjusted OR [AOR] =  (AOR = 1.78, 95% CI 1.06, 2.99; *p* < *0.01*) lacking formal education (AOR = 3.67, 95% CI 1.48–9.09; *p* < *0.01*), and attending primary education (AOR = 2.23, 95% CI 1.05–2.32; *p* < *0.01*) were significantly associated with VL non-suppression. Also, relative to age; older adolescents aged 16–19 (AOR = 1.78, 95% CI 1.06, 2.99; *p* < *0.05*), and those with WHO clinical stage II (AOR = 0.48, 95% CI 0.28, 0.82; *p* < *0.01*), had higher odds of viral non-suppression. Longer duration on ART for > 5 years (AOR = 3.47, 95% CI 1.39–8.66; *p* < *0.01*), and > 12 months to < 5 years (AOR = 3.20, 95% CI 1.31–7.82; *p* < *0.05*), being on second line ART regimen (AOR = 2.38, 95% CI 1.53–3.72*; p* < *0.001*) and having comorbidities (AOR = 3.28, 95% CI 1.20–9.00; *p* < *0.05*) all remained significantly associated with VL non-suppression in the adjusted model (Table 2).

## Discussion

In this cross-sectional study of 567 HIV-infected adolescents on ART in rural Uganda, in which the mean age was 15.6 years, we found that 31.7% of the adolescents had viral non-suppression during the study period. Being male, age 16–19 years, lack of formal education, primary education level, duration on ART therapy for > 12 months to ≤ 5 years and > 5 years, WHO Clinical Staging II, second-line ART regimen, and presence of comorbidities were significantly associated with viral non-suppression.

Our finding of a VL non-suppression rate of 31.4% found in this study is almost comparable to the national age-specific rate of 27.5% among adolescents in 2017/2018 [18]. A cross-sectional study by Natukunda et al. in Uganda [8] found the VL non-suppression prevalence among adolescents to be 34.5%, which is a little higher than this study’s rate. This study’s rate is also higher than that reported for low- and middle-income countries (29%) and a lot higher than that reported in South Africa (19%) [22]. Adolescents in Uganda, therefore, show a higher rate of viral non-suppression and are lagging behind other age groups in achieving the 95–95–95 targets, making them a weak link in the collective effort to reach the goal above [18]. The possibility of having good adherence yet with poor viral outcomes cannot be ruled out in a setting like Uganda where treatment options are not regularly updated and there is limited routine ART resistance testing [4]. Besides only reporting the proportion of adolescents with non-suppressed viral loads, the present study also identified independent predictors of viral non-suppression among this critical population. The findings in the study suggest the need for HIV clinical services to design and implement targeted interventions with a particular focus on male gender, co-morbidities, adolescents on second line ART and those on longer duration of ART.

The generally poorer adherence to ART among males is possibly related to a “background reluctance to access healthcare” and might lead to a higher likelihood of viral non-suppression [23]. The results are consistent with a cross-sectional study conducted in Malawi [24], a Mozambican case–control study [25], and an observational cohort study [26] in Cameroon where more males were non-suppressed than females. However, a study in Tanzania found that the female gender was independently associated with VL non-suppression [27].

Having a comorbidity, such as tuberculosis in most of the cases, increased the odds of being virally non-suppressed. The pill burden among such adolescents might probably explain the poor adherence and the subsequent non-suppression. Also, drug-to-drug interaction may affect the efficacy of ARVs, leading lead to non-suppression [28, 29]. These findings are in line with a study in Zimbabwe and in South Africa, which showed that adolescents on prolonged medication for another disease were likelier to be virally non-suppressed [28, 29].

Additionally, being on the second-line of ART increased the odds of being virally non-suppressed. Many adolescents on second-line regimens likely have a history of treatment disturbances like poor adherence, leading to virologic failure, and a switch to second-line. A study found that deficient adherence to first-line ART portended suboptimal adherence to second-line ART [30]. A retrospective cross-sectional study in Ethiopia [31] and a case–control study in Eswatini [32] found that EFV-based ART was more likely cause to VL suppression compared to NVP-based regimens.

Furthermore, the longer the adolescents were on ART, the higher their odds of VL non-suppression. It is plausible that since longer ART duration is typical of perinatally-infected adolescents, non-suppression may be a result of drug resistance over time, leading to non-suppression or treatment fatigue leading to reduced adherence to ART [8]. A systematic review [33] showed that longer ART duration was associated with higher levels of acquired ART resistance. This is consistent with studies in Uganda [8] and in Swaziland [35], where VL load non-suppression was higher among adolescents on ART for 5 years and above. However, a study in South Africa found that adolescents on ART between 6 months and one year were more likely to be non-suppressed compared to those on treatment for more extended periods [36].

Regarding older age and VL non-suppression, it is conceivable that because older adolescents usually have been on ART for longer periods, they suffer from medication fatigue and miss appointments, leading to poorer adherence [31, 37]. Additionally, older adolescents are in a complex, transitory life period, associated with an inability to make important life decisions, and more sensitivity to stigma, leading to poorer outcomes [38]. This finding was consistent with cross-sectional studies in Uganda [13] and Zimbabwe [38] which showed that older adolescents were likelier to be non-suppressed compared to younger adolescents. However, a retrospective study in Kenya [39] and a prospective cohort study in Tanzania [27] found no association between VL non-suppression and age; and that older adolescence was associated with VL non-suppression, respectively. In this study, because of the age disaggregation employed in Uganda where ages 0–14 are considered children while those 16–19 are considered adults [34], we found it appropriate to use similar disaggregation, thus the 16 years old cut off.

Also, adolescents in higher clinical stages likely have a history of treatment disturbances, which may continue and lead to increased occurrence of VL non-suppression [40]. A study in Ethiopia [31] also showed an increased likelihood of viral non-suppression in clinical stage II compared stage I, while another in Uganda [34] found no association between clinical stage and VL non-suppression. While a study in Swaziland [37] and another in Nigeria [41] found that stage 3 or stage 4 was significantly associated with VL non-suppression, the very few adolescents in stage 3 or 4 might explain why the analysis did not reveal any association between these stages and VL non-suppression in this study. Nevertheless, the findings are more-or-less congruent with the above-stated studies and point to a direct relationship between increasing clinical stage and VL non-suppression.

Lastly, less the education the adolescents had, the higher their odds of VL non-suppression. It is potentially because the more educated adolescents appreciated more the importance of taking medication, and these also seemed to have more parental support compared to the uneducated [42]. The findings are in line with a cross-sectional study in Ethiopia, where uneducated participants were more likely to be non-suppressed compared to educated ones [42]. However, a study in Uganda found that adolescents who attended school, especially higher school, had inferior ART adherence and this was attributed to the stigma and lack of privacy in a school setting for poor adherence and outcomes [43].

The strengths of this cross-sectional study include the use of five high-volume HIV programmes scattered across the district and use of probability proportional to size sampling to identify adolescents thus, making the sample more representative of adolescents in Eastern Uganda, and, subsequently, increasing the external validity of the study [44]. The major limitation of this study was the usage of routine data that was no collected for research purposes, which handicapped the researcher as regards the exposure variables that could be studied. Our limitation was that we did not analyze data on variables like household income or distance to the health facilities that would have allowed us to control for more concealed demographic confounders, because they are not routinely collected in public health facilities. Lastly, the cross-sectional design did not also allow for analysis of time-varying covariates.

## Conclusion

In conclusion, we found that adolescents in Mbale District, continue to face worse HIV viral load outcomes with almost one-third of adolescents in Mbale District virally non-suppressed, a prevalence higher than the national average. The determinants of viral load non-suppression among adolescents are unique and thus require unique interventions. Urgent adolescent-friendly strategies to decrease non-suppression rates such a peer involvement, VL focal persions to identify VL non-suppression and actively follow up with these adolescents, patient education on VL non-suppression, and demand creation for ART especially for newly initiated adolescents and adolescents on ART for protracted periods, should be laid with these determinants in mind so that they (strategies) are targeted, if efforts to achieve HIV epidemic control in resource-limited settings are to be successful.

## Data Availability

The dataset used and analyzed during this study is available from the corresponding author on reasonable request.

## Abbreviations

AIDS: Acquired Immune Deficiency Syndrome
ART: Anti-retroviral treatment
DHO: District Health Officer
HIV: Human Immunodeficiency Virus
IAC: Intensified Adherence Counselling
MoH: Ministry of Health
PLHIV: People Living with HIV
UAC: Uganda AIDS Commission
UBOS: Uganda Bureau of Statistics
UNAIDS: Joint United Nations Programme on HIV/AIDS
USAID: United States Agency for International Development
VL: Viral Load
WHO: World Health Organization
